# Comparative Agreement of Intradermal Tuberculin Test and Interferon-Gamma Release Assay in Bovine Tuberculosis Surveillance in Sicily, Italy (2024–2025)

**DOI:** 10.3390/vetsci13090849

**Published:** 2026-08-22

**Authors:** Delia Gambino, Lucia Galuppo, Tiziana Orefice, Maurilio Saladino, Giuseppe Barbaccia, Natale Sergio Glorioso, Antonino Calagna, Mario Richiusa, Antonio Vella, Giovanni Cassata, Francesca Di Gaudio

**Affiliations:** 1 Istituto Zooprofilattico Sperimentale della Sicilia “A. Mirri”, Via G. Marinuzzi, 3, 90129 Palermo, Italy; delia.gambino@izssicilia.it (D.G.); tiziana.orefice@izssicilia.it (T.O.); maurilio.saladino@izssicilia.it (M.S.); giuseppe.barbaccia@izssicilia.it (G.B.); antonio.vella@izssicilia.it (A.V.); giovanni.cassata@izssicilia.it (G.C.); 2 Department of Agricultural, Food and Forestry Science (SAAF), Università di Palermo, Viale delle Scienze 13, 90128 Palermo, Italy; 3 Dipartimento di Prevenzione Veterinario, Azienda Sanitaria Provinciale Palermo, Via C. Onorato, 6, 90129 Palermo, Italy; natalesergio.glorioso@asppalermo.org (N.S.G.); antonino.calagna@asppalermo.org (A.C.); mariorichiusa@asppalermo.org (M.R.)

**Keywords:** bovine tuberculosis, intradermal tuberculin test (IDT), interferon-gamma assay (IFN-γ), diagnostic agreement, endemic area

## Abstract

Bovine tuberculosis (bTB) remains endemic in southern Italy, particularly in Sicily, posing challenges for disease control and eradication. This study evaluated the diagnostic agreement between the intradermal tuberculin test (IDT), interferon-gamma release assay (IFN-γ), and post-mortem lesion detection in 1801 cattle from 44 herds in the province of Palermo during 2024–2025. Diagnostic agreement between IDT and IFN-γ was substantial in 2024 (κ = 0.731; 95% CI: 0.64–0.82; n = 991) and decreased to moderate in 2025 (κ = 0.453; 95% CI: 0.33–0.57; n = 572), reflecting a higher proportion of IFN-γ positive and inconclusive animals. An increase in inconclusive results for IFN-γ was observed in 2025, but the available data did not allow investigation of the factors underlying this difference. Agreement between ante-mortem tests and post-mortem lesion detection was poor in a selected subgroup of slaughtered animals, potentially subject to verification bias, highlighting the limitations of single diagnostic tools and the need for a complementary approach combining immunological and pathological methods. These findings support the interpretation of the complementary roles of IDT and IFN-γ within herd-level bTB surveillance, highlight the need for standardized protocols to manage inconclusive IFN-γ results, and underline the complementary contribution of post-mortem inspection within integrated surveillance strategies in endemic settings. Official confirmation of bovine tuberculosis relies on an integrated evaluation combining diagnostic results, post-mortem findings, and epidemiological investigations. Given the absence of a validated gold standard applicable to all animals in this observational dataset, the study was designed as a diagnostic agreement study rather than a formal accuracy assessment.

## Simple Summary

Bovine tuberculosis (bTB) is a contagious disease that affects cattle, threatens animal health, and causes significant economic losses worldwide. Despite long-standing eradication programs, the disease remains a persistent problem in some regions of southern Italy, including Sicily. Reliable diagnostic tools are essential to identify infected animals as early as possible and prevent disease spread within and between herds. In this study, we compared the results of the two main tests used during routine surveillance—the intradermal tuberculin skin test and the interferon-gamma release assay—and examined how their results relate to findings observed after slaughter. Our study highlights the strengths and limitations of each diagnostic approach when applied under real field conditions in an endemic area. The findings reinforce the importance of combining different diagnostic methods rather than relying on a single test and emphasize the need for clear procedures to manage uncertain test results. These results provide useful information for veterinary authorities and policymakers seeking to improve bovine tuberculosis surveillance and strengthen disease control and eradication programs.

## 1. Introduction

Bovine tuberculosis (bTB), caused by *Mycobacterium bovis*, is a chronic zoonotic disease of major economic and public health relevance, resulting in substantial losses due to the culling of infected animals, trade restrictions, and the long-term costs associated with control and eradication programmes, despite the long-standing implementation of European Union control measures [1,2].

At the EU level, Regulation (EU) 2016/429 and its delegated act, including Commission Delegated Regulation (EU) 2020/689, define harmonized rules for surveillance, control, and eradication of listed diseases, including bTB, and set criteria for disease-free status [2]. In Italy, these rules are implemented through the Ministry of Health Decree of 2 May 2024, establishing mandatory eradication programmes for bovine, ovine-caprine brucellosis and bovine/buffalo tuberculosis [3]. These provisions establish a surveillance framework based on ante-mortem testing, complemented by post-mortem inspection of animals slaughtered under the eradication programme or routine procedures.

Under the Italian bTB eradication programme, animals with non-negative results to official ante-mortem tests are managed according to the procedures established by national legislation. Depending on the results of the diagnostic and epidemiological investigations, animals may be classified as suspected or confirmed cases of MTBC infection. Confirmed infected animals are subject to removal from the herd and compulsory culling, while suspected animals may also be sent for slaughter for further investigation and laboratory confirmation [3]. Despite overall reductions in prevalence in non-tuberculosis-free regions from 0.20–0.23% in 2018–2019 to 0.14–0.15% in 2021–2022, endemic clusters persist, mainly in southern Italy, including Sicily (34.9% of outbreaks in 2022) [4,5]. Cattle remain the primary reservoir, while wildlife, particularly wild boar (Sus scrofa), may act as maintenance hosts in specific ecological contexts [6].

In southern Italy, and particularly in Sicily, bTB remains highly endemic, with apparent prevalence during routine screening ranging between 7% and 10%, and estimates of true prevalence adjusted for diagnostic test performance approaching 10% [7,8,9]. The epidemiological pattern is highly complex, driven by multi-host interactions, intense cattle movements, and heterogeneous farm management practices, which significantly hinder the design and implementation of effective control strategies [1]. Current control measures rely mainly on routine surveillance using the indirect tuberculin skin test (IDT), which is characterized by high specificity but limited sensitivity, particularly in cases of latent infection or in anergic animals. The integration of IDT with the indirect interferon-gamma release assay (IFN-γ) allows earlier detection of infection and improves overall herd-level sensitivity when applied in parallel testing schemes, as recommended in high-risk and endemic settings [3,10,11]. Post-mortem surveillance, including the detection of tuberculosis-compatible lesions and, when performed, subsequent bacteriological or molecular investigations, provides complementary information within the surveillance system [2,3]. However, lesion detection alone is not pathognomonic and cannot establish definitive etiological confirmation. Although the performance of IDT and IFN-γ has been extensively investigated, less information is available on their agreement when both tests are applied under routine surveillance conditions, particularly in endemic settings where discordant and inconclusive results may complicate the interpretation of diagnostic outcomes.

In this context, the present study evaluates the agreement between IDT and IFN-γ assay under routine bTB surveillance conditions in an endemic area of southern Italy, while also exploring the interpretation of inconclusive IFN-γ results and the limitations of agreement analyses involving post-mortem lesion detection. The study is based on data jointly collected by the Provincial Health Authority—Veterinary Prevention Department (Azienda Sanitaria Provinciale Palermo, Dipartimento di Prevenzione Veterinaria) and the Istituto Zooprofilattico Sperimentale della Sicilia (IZS Sicilia, Palermo). The dataset refers to bTB surveillance activities conducted in the province of Palermo (Sicily, Italy) during the period 2024–2025, coinciding with the implementation of the Italian Ministry of Health Decree of 2 May 2024, which updated operational procedures within the national eradication programme. This provided an opportunity to evaluate diagnostic agreement under routine surveillance conditions following the implementation of the revised national framework, while also examining how inconclusive results and the selective availability of post-mortem data affect the interpretation of agreement estimates in an endemic setting. By addressing these issues using routine programme data rather than data collected under controlled experimental conditions, the study provides field-based evidence on the interpretation and limitations of diagnostic agreement measures in endemic bTB surveillance. The primary objective of this study was to assess the pairwise agreement between the intradermal tuberculin test (IDT) and the IFN-γ assay in cattle from an endemic area of southern Italy. Secondary objectives were (i) to explore the implications of diagnostic agreement for the interpretation of discordant and inconclusive results, with particular attention to the occurrence of inconclusive IFN-γ results, and (ii) to explore the agreement between ante-mortem test results and post-mortem lesion detection, while examining the methodological limitations associated with the selective availability of post-mortem data within routine bTB surveillance.

## 2. Materials and Methods

### 2.1. Study Design, Area and Population

A retrospective observational study was conducted to evaluate the pairwise diagnostic agreement among bTB diagnostic tests in the province of Palermo (Sicily, Italy), a region with persistent endemicity.

The study included cattle belonging to herds previously classified as suspected and/or confirmed bTB outbreaks, located in the province of Palermo and enrolled in the national bTB eradication programme during the study period (January 2024–December 2025). The area is characterized by heterogeneous production systems, frequent animal movements, and potential interactions with wildlife reservoirs, which may influence local transmission dynamics. Eligibility criteria were defined at both herd and animal level and are summarized in Table 1.

**Figure 1:**
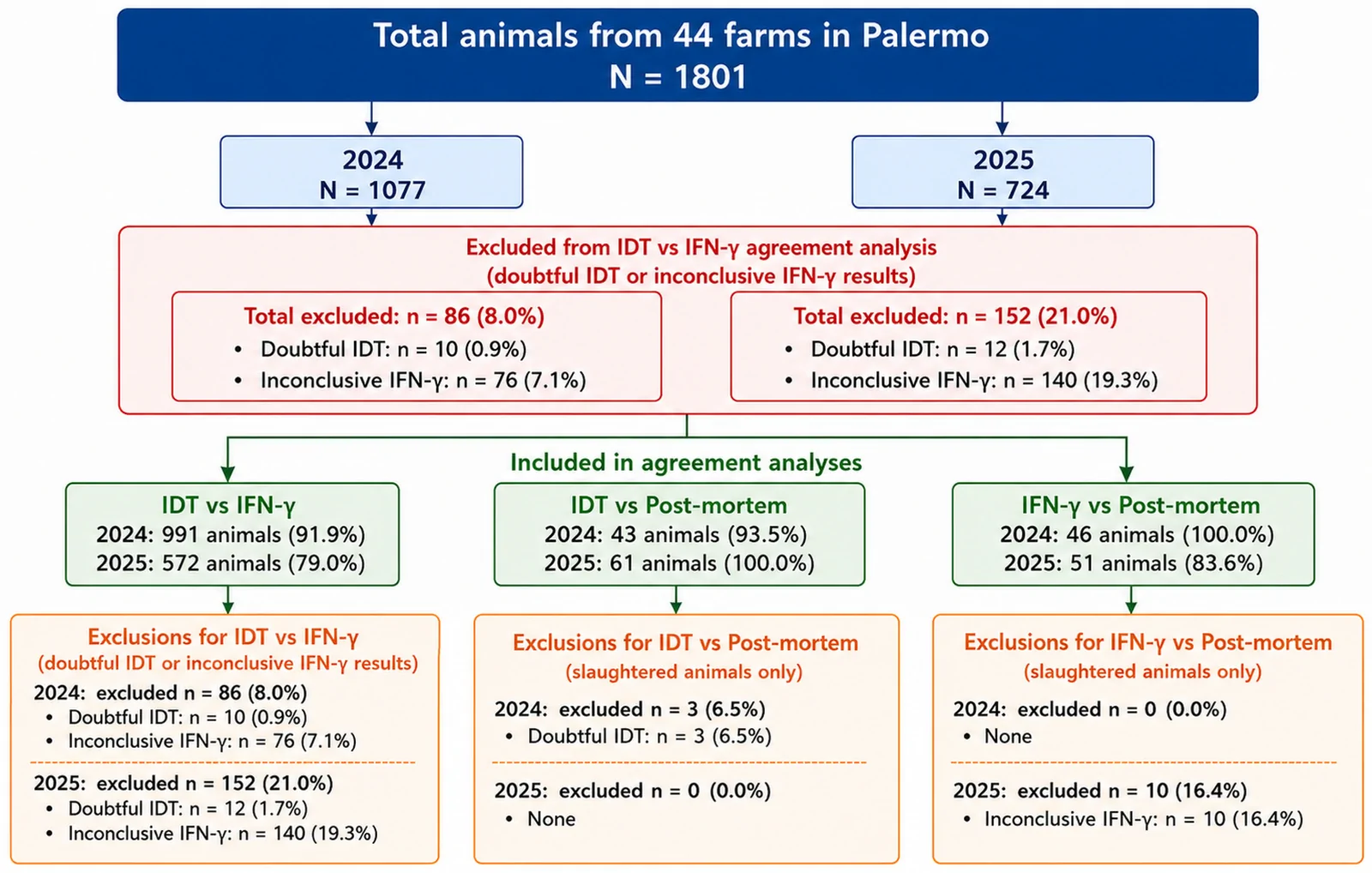
STROBE-compliant flow diagram showing the inclusion and exclusion of cattle in this study. The diagram illustrates the total number of animals enrolled, the distribution by study year, and the number and reason for exclusions from each pairwise agreement analysis. Animals with doubtful IDT results or inconclusive IFN-γ results were excluded from the relevant comparisons. For post-mortem comparisons, only slaughtered animals with available post-mortem data were included. The final numbers of animals included in the IDT vs. IFN-γ, IDT vs. post-mortem lesion detection, and IFN-γ vs. post-mortem lesion detection analyses are reported separately for 2024 and 2025.

During the study period, the herds were subjected to the official bovine tuberculosis (bTB) eradication measures set out in the Ministerial Decree of 2 May 2024, including routine annual testing, outbreak investigations and follow-up testing on previously infected herds. Animals meeting the inclusion criteria and not fulfilling any exclusion criteria were retained for analysis. After application of these criteria, a total of 1801 animals were included in the final dataset. No formal a priori sample size calculation was performed, as this retrospective study was based on all eligible animals with available surveillance data during the predefined study period. The overall sample size therefore reflects routine programme implementation rather than an experimental design with predefined statistical power assumptions. While the main IDT–IFN-γ agreement analysis was based on the full eligible population, analyses involving post-mortem findings were restricted to a substantially smaller subgroup of slaughtered animals. Consequently, these subgroup estimates were expected to have lower statistical precision and were interpreted with caution. A STROBE-compliant flow diagram illustrating the selection process is presented in Figure 1.

### 2.2. Data Collection

The data were collected jointly by the Provincial Health Authority—Veterinary Prevention Department (ASP Palermo, Veterinary Prevention Department) and the Istituto Zooprofilattico Sperimentale della Sicilia (IZS Sicilia), Palermo.

The IDT results, including individual animal identifiers, herd codes, test dates, and diagnostic classifications, were retrieved from the VETINFO national veterinary information system through the data management platforms of the Palermo Local Health Authority (ASP Palermo) [12]. IFN-γ test results and associated laboratory metadata were retrieved from the Laboratory Information System (SILAB) of the IZS Sicilia.

Post-mortem data were obtained from official slaughterhouse inspection reports drawn up by ASP Palermo veterinarians.

Datasets were shared in structured spreadsheet format and manually merged using the individual animal identification code as the unique linking variable. Each record was verified individually to ensure correct matching and resolve discrepancies before statistical analysis. Animals with missing or unverifiable records were excluded, as detailed in Figure 1.

### 2.3. Diagnostic Procedures

All animals included in the study underwent routine diagnostic procedures according to the Decree of 2 May 2024 of the Italian Ministry of Health and the protocols of national and European reference laboratories [3,11].

The IDT tests were carried out by official veterinarians from the Palermo ASP, by injecting 0.1 mL of purified bovine protein derivatives (PPD) into the cervical region. Skin thickness was measured before inoculation and 72 h after the injection. Animals were classified as negative (bovine PPD reaction ≤ 2 mm), doubtful (bovine PPD reaction 2–4 mm with no clinical signs), or positive (bovine PPD reaction > 4 mm), in accordance with Commission Delegated Regulation (EU) 2020/689 and current national guidelines [2,3,10].

IFN-γ assays were performed at the IZS Sicilia using the Bovigam^®^ kit (Thermo Fisher Scientific, Milan, Italy), following the manufacturer’s instructions and guidelines from the national and European reference laboratories. Whole blood samples were collected by ASP Palermo official veterinarians and transported to IZS Sicilia for processing within 8 h of collection. Samples were stimulated with bovine tuberculin (bPPD), avian tuberculin (aPPD), a negative control (nil, PBS), and a positive mitogen control, and optical density (OD) values were measured at 450 and 620 nm after overnight incubation. Results were interpreted as follows: positive when OD (bPPD − nil) ≥ 0.1 and OD (bPPD − aPPD) ≥ 0.1; negative when OD (bPPD − nil) < 0.1 or OD (bPPD − aPPD) < 0.1, provided the mitogen control yielded a valid response; and inconclusive when the result met the criteria for a negative classification but the mitogen control also failed to elicit an adequate response, indicating potential lymphocyte non-reactivity and precluding reliable result interpretation [13].

Post-mortem inspections were carried out by the Veterinary Services of the Health Authorities at slaughterhouses through an inspection in accordance with current regulations (EU Implementing Regulation 2019/627; Ministerial Decree of 2 May 2024; regional guidelines) to identify any lesions consistent with bTB. Not all study animals were slaughtered during the study period; post-mortem data are therefore available only for the subset of animals that reached slaughter.

### 2.4. Statistical Analysis

Descriptive statistics were performed to summarise data generated within the bTB surveillance programme. Absolute frequencies and proportions were calculated for IDT results (positive, negative, doubtful), IFN-γ assay results (positive, negative, inconclusive), and when available, post-mortem lesion status (presence/absence). All analyses were stratified by study year (2024 and 2025).

Agreement between diagnostic tests was assessed using three complementary metrics: (i) crude observed agreement (Po); (ii) Cohen’s kappa coefficient (κ), which quantifies agreement beyond chance; and (iii) the prevalence- and bias-adjusted kappa (PABAK), which corrects for the kappa paradox observed in populations with markedly skewed prevalence. For each pairwise comparison, 2 × 2 contingency tables were constructed after exclusion of non-definitive test outcomes to allow binary classification. Specifically, animals with doubtful IDT results (2024: 10; 2025: 12) were excluded from analyses involving IDT, and animals with inconclusive IFN-γ results (2024: 76; 2025: 140) were excluded from analyses involving IFN-γ. This approach was adopted to ensure binary classification for kappa estimation and is consistent with standard practice in diagnostic agreement studies where non-definitive outcomes are considered separately from the primary analysis. Inconclusive results were not reclassified as either positive or negative, as neither assumption is biologically justified: inconclusive IFN-γ results reflect lymphocyte non-reactivity rather than a true diagnostic category, and arbitrary reclassification would introduce misclassification bias of uncertain direction.

For comparisons with post-mortem lesion status, only slaughtered animals with complete ante-mortem test results were included. The final numbers of animals included in each comparison were 991 and 572 for IDT vs. IFN-γ in 2024 and 2025, respectively; 43 and 61 for IDT vs. lesions; and 46 and 51 for IFN-γ vs. lesions. The relatively small number of animals included in post-mortem comparisons (n = 43–61) resulted in limited statistical precision. Therefore, 95% confidence intervals were considered essential for interpreting the uncertainty surrounding these subgroup agreement estimates, and the analyses involving post-mortem data should be interpreted with caution.

Expected agreement (Pe) was calculated from the marginal totals of each contingency table. Cohen’s kappa was estimated using the standard formula: κ = (Po − Pe)/(1 − Pe). The 95% confidence intervals for κ were calculated using the standard error derived from the contingency tables according to Fleiss [14]. PABAK was calculated as: PABAK = 2 × Po − 1. Agreement strength was interpreted according to the criteria proposed by Landis and Koch, with explicit thresholds (e.g., <0 poor, 0–0.20 slight, 0.21–0.40 fair, 0.41–0.60 moderate, 0.61–0.80 substantial, 0.81–1 almost perfect) [15].

Given that slaughtered animals may not constitute a random sample of the tested population, potential verification bias was assessed by comparing the proportion of IDT- and IFN-γ-positive animals among slaughtered cattle with the corresponding proportions in the overall tested population, stratified by study year [16]. Marked differences were interpreted as indicative of non-random selection for slaughter.

All analyses were conducted separately for each study year using RStudio (Version 2026.01.0+392, Posit PBC, Boston, MA, USA), with the dplyr, tidyr, epitools, vcd, irr, fmsb, ggplot2, and ggpubr packages for data management and agreement analyses (Version 2026.01.0+392, Posit PBC, Boston, MA, USA).

## 3. Results

### 3.1. Descriptive Results

A total of 1801 animals from 44 cattle farms in the province of Palermo (Sicily, Italy) were included in the study. Of these, 1077 animals were tested in 2024 and 724 in 2025.

The distribution of IDT results, IFN-γ assay outcomes, and post-mortem findings is summarized in Table 2.

Of the 1801 animals tested ante-mortem, 107 (5.9%) were slaughtered during the study period and had post-mortem data available (46 in 2024 and 61 in 2025). Among these slaughtered animals, 34 of 107 (31.8%) were IDT-positive, compared with 5.2% (93/1801) in the full tested population. IFN-γ positivity was 50.5% (54/107) among slaughtered animals versus 9.1% (163/1801) in the overall cohort. When stratified by year, IDT positivity among slaughtered animals was 34.8% (16/46) in 2024 and 29.5% (18/61) in 2025, compared with 5.3% and 5.0% in the respective annual populations. IFN-γ positivity was 56.5% (26/46) in 2024 and 45.9% (28/61) in 2025, compared with 7.3% and 11.6% in the full tested population for the same years.

The IDT positivity rate remained broadly stable between 2024 and 2025 (5.3% and 5.0%, respectively). In contrast, the IFN-γ test showed an increase in the proportion of positive animals, rising from 7.3% in 2024 to 11.6% in 2025.

A higher proportion of inconclusive IFN-γ results was observed in 2025 (19.3% vs. 7.1% in 2024). The available dataset did not include sufficient individual-level clinical, epidemiological, or temporal variables to support multivariable or hierarchical modelling of factors associated with inconclusive results. Therefore, no formal analysis of potential predictors was performed, and no inference regarding the causes of this increase can be drawn.

### 3.2. Agreement Analyses

Only animals with complete and definitive results for the diagnostic tests included in each comparison were considered in the pairwise agreement analyses. Animals with doubtful IDT or inconclusive IFN-γ results were excluded from the relevant comparisons. The final numbers of animals included in each comparison were 991 and 572 for IDT vs. IFN-γ in 2024 and 2025, respectively; 43 and 61 for IDT vs. lesions; 46 and 51 for IFN-γ vs. lesions; and 43 and 52 for IDT vs. IFN-γ within the slaughtered subgroup.

Agreement between diagnostic tests is summarised in Table 3. The contingency tables underlying all pairwise agreement analyses are presented in Supplementary Table S1.

Observed agreement between IDT and IFN-γ was high in both study years (Po = 0.967 in 2024 and 0.909 in 2025), whereas Cohen’s kappa indicated substantial agreement in 2024 (κ = 0.731; 95% CI: 0.640–0.821) and moderate agreement in 2025 (κ = 0.453; 95% CI: 0.311–0.595). PABAK values were 0.933 and 0.818, respectively. The greater divergence between κ and PABAK in 2025 occurred in the context of an unequal marginal distribution of test results, associated with the higher proportion of IFN-γ-positive animals.

Agreement between ante-mortem tests and post-mortem lesion detection was assessed in the slaughtered subgroup. Cohen’s kappa values were close to zero or negative in all comparisons (IDT: κ = −0.073 in 2024, κ = −0.086 in 2025; IFN-γ: κ = 0.057 in 2024, κ = −0.039 in 2025), with 95% confidence intervals that included both negative and positive values. Given the limited number of animals and the width of these intervals, the point estimates should be interpreted cautiously and do not allow reliable conclusions regarding the direction or magnitude of agreement between ante-mortem test results and post-mortem lesion detection in this subgroup.

In the slaughtered subgroup, agreement between IDT and IFN-γ was poor in both years (2024: κ = −0.045, 95% CI: −0.130 to 0.040, PABAK = 0.349; 2025: κ = −0.039, 95% CI: −0.115 to 0.037, PABAK = −0.231). These estimates are not meaningfully interpretable due to a highly unbalanced distribution of IFN-γ results in this selected population (42/43 in 2024; 51/52 in 2025), and are reported for completeness only (see Table 3, note †).

## 4. Discussion

This study evaluated the diagnostic agreement between the IDT and IFN-γ assays, and post-mortem lesion detection in 1801 cattle from 44 herds in the province of Palermo, Sicily, during 2024–2025. The findings provide insight into the limitations and complementarity of ante-mortem and post-mortem diagnostic approaches in a high-prevalence, endemic setting.

Diagnostic agreement between IDT and IFN-γ varied between the two study years. The divergence between κ and PABAK reflects the known sensitivity of Cohen’s kappa to prevalence imbalance, which may underestimate agreement when positive results are unevenly distributed [17]. Thus, the lower κ observed in 2025 may partly reflect changes in the distribution of test outcomes rather than reduced diagnostic performance. In contrast, PABAK remained high after accounting for prevalence effects, suggesting that the discrepancy between the two measures was largely influenced by the marginal distribution of results. However, the available data do not allow us to determine whether biological or technical factors also contributed to the observed year-to-year differences. These findings highlight the importance of interpreting agreement measures cautiously in endemic settings with unequal prevalence. High observed agreement should be interpreted alongside the distribution of concordant positive and negative results, as a predominance of concordant negative outcomes may contribute substantially to Po.

The higher proportion of IFN-γ positive animals observed in 2025 indicates a greater number of positive results compared with IDT. This difference may be consistent with the different biological and analytical characteristics of the two tests; however, the present study was designed to assess diagnostic agreement and does not allow conclusions regarding their relative diagnostic sensitivity or accuracy [18].

The marked increase in inconclusive IFN-γ results in 2025 represents an interesting finding of the present study. However, the available dataset did not contain sufficient individual-level clinical, epidemiological, or temporal information to formally investigate factors associated with inconclusive outcomes using multivariable or hierarchical models. Therefore, the observed increase can only be described, and its underlying determinants cannot be inferred from the present data. Potential biological or environmental factors reported in previous studies may provide hypotheses for future investigations but cannot be considered explanatory factors in the present study [19,20]. This finding also underscores the operational importance of clear protocols for repeat testing and interpretation of inconclusive results, including defined retesting intervals and herd-level decision criteria.

Post-mortem diagnostic agreement analyses yielded unstable estimates with wide confidence intervals, precluding a reliable assessment of the actual agreement between ante-mortem immunological tests and post-mortem lesion detection. This uncertainty reflects not only limited sample size but also the highly selected nature and imbalanced distribution of outcomes within the slaughtered subgroup. Additional resampling or modelling approaches would not overcome these limitations or restore the information absent from the observed data. It is important to emphasize that this limitation highlights a methodological challenge inherent in routine programmes for the eradication of bovine tuberculosis (bTB): as slaughter is generally determined by positive ante-mortem test results, rather than by random sampling, the validation of ante-mortem tests against post-mortem findings is inherently limited. An exception is made for animals slaughtered under routine conditions in which lesions consistent with bTB are unexpectedly identified. Therefore, reliable estimates of the agreement between test results and lesions require dedicated slaughter cohorts, selected independently of the results of diagnostic tests. The marked enrichment of test-positive animals among slaughtered cattle illustrates why such analyses must be interpreted with extreme caution [16]. This enrichment of test-positive animals means that the slaughtered subgroup had a markedly different distribution of diagnostic outcomes from the overall surveillance population. Consequently, agreement estimates involving post-mortem lesions may have been distorted by the non-random verification process and cannot be generalized to the full tested population. In particular, the strong imbalance in test outcomes within the slaughtered subgroup may contribute to unstable kappa estimates, making the observed direction and magnitude of agreement difficult to interpret. Furthermore, post-mortem lesion detection is not a perfect reference standard: lesions may be absent or undetectable in early or paucibacillary infections, and diagnostic sensitivity depends on inspection procedures and operator expertise [21].

Discordance may also reflect biological factors, including reduced IDT reactivity in anergic animals, variability in IFN-γ responses associated with infection stage, stress, or seasonal immunomodulation [22,23,24]. Taken together, these considerations confirm that ante-mortem and post-mortem approaches provide complementary rather than interchangeable information, and that integrated diagnostic strategies combining immunological and pathological evidence remain essential in endemic settings. Future studies aiming to evaluate the sensitivity of ante-mortem tests against a post-mortem reference should prospectively enrol animals for slaughter independently of their test result, following a pre-specified sampling design.

The present findings have direct implications for bTB control policies in endemic settings. Parallel use of IDT and IFN-γ, as recommended by European and national guidelines for high-risk areas [2,3,10], reflects the complementary information provided by these ante-mortem approaches within routine surveillance. Post-mortem inspection remains an important component of the surveillance and confirmation process and provides information that cannot be obtained from ante-mortem testing alone. Integrated surveillance systems combining immunological, pathological, and bacteriological evidence are particularly important in endemic regions characterized by persistent multi-host circulation of *M. bovis* [3,5].

This study is strengthened by the large multi-herd dataset integrating ante-mortem and post-mortem data with complementary agreement metrics, providing a comprehensive picture of bTB diagnostic agreement in an endemic setting. Nonetheless, several limitations should be acknowledged: the fact that analyses were conducted at the individual animal level without accounting for clustering within herds, which may affect the precision of the agreement estimates and their confidence intervals; the absence of systematic bacteriological confirmation across all animals; the lack of molecular typing data, which precluded epidemiological assessment of transmission dynamics and strain diversity; the lack of information on potentially relevant animal- and herd-level factors, including age, sex, breed, herd characteristics, and vaccination history, which prevented adjustment for potential confounding; the limited number of animals undergoing post-mortem examination, which resulted in wide confidence intervals for κ estimates and reduced statistical precision in subgroup analyses, thereby limiting the strength of conclusions regarding agreement between ante-mortem tests and post-mortem lesion detection; and the exclusion of inconclusive IDT and IFN-γ results from agreement analyses. In 2025, inconclusive IFN-γ results represented 19.3% of all outcomes, and their exclusion may have introduced selection bias if these animals differ systematically from those with definitive results. Therefore, the reported agreement estimates should be interpreted as applying only to animals with definitive results and may not be fully generalizable to the entire surveillance population. A sensitivity analysis reclassifying inconclusive results as positive or negative was not performed, as no biologically grounded threshold exists to guide such reclassification in this dataset. Future studies should prospectively define criteria for managing inconclusive results before data collection, ideally with follow-up testing to resolve indeterminate outcomes. In addition, the relatively small number of animals undergoing post-mortem examination resulted in limited precision of the corresponding agreement estimates. Therefore, findings from this subgroup should be interpreted as exploratory and should not be considered definitive evidence of the magnitude of agreement between ante-mortem tests and post-mortem lesion detection. Despite these constraints, the study provides a solid epidemiological basis for evaluating and improving integrated diagnostic approaches to bTB control in endemic cattle populations.

## 5. Conclusions

This study assessed the diagnostic agreement between IDT and IFN-γ and explored their agreement with post-mortem lesion detection in cattle from an endemic area of southern Italy, contributing field-based evidence on the interpretation and limitations of diagnostic agreement under routine bTB surveillance.

Diagnostic agreement between IDT and IFN-γ ranged from substantial to moderate across the two study years, highlighting their complementary role within the current surveillance framework. The observed differences highlight the importance of interpreting agreement measures in relation to the distribution of test results, particularly in the presence of discordant and inconclusive outcomes. The marked increase in inconclusive IFN-γ results observed in 2025 underscores the need for standardised protocols governing the management of non-definitive outcomes, including defined retesting intervals and herd-level decision criteria.

The post-mortem analysis should be considered exploratory because only a selected subgroup of animals underwent examination, resulting in substantial verification bias. Moreover, lesion detection cannot be considered a definitive reference standard in the absence of systematic bacteriological or molecular confirmation. Post-mortem lesion detection provides complementary rather than interchangeable information relative to ante-mortem immunological testing; meaningful agreement estimation between these approaches requires prospective study designs with unselected slaughter cohorts, which routine surveillance data cannot support.

Integrated diagnostic strategies combining IDT, IFN-γ, and post-mortem inspection remain relevant within routine bTB surveillance in endemic areas. Future research should address the identified methodological gaps through prospective designs with systematic bacteriological confirmation and pre-specified criteria for managing inconclusive test results.

## Figures and Tables

**Table 1:** Eligibility criteria applied at herd and animal level.

Level	Inclusion Criteria	Exclusion Criteria
Herd	Located in the province of Palermo Subjected to official bTB surveillance between January 2024 and December 2025 (routine annual testing, outbreak investigation, or follow-up testing)	Herds outside the study area Herds not officially enrolled in the eradication programme during the study period
Animal	Belonging to an eligible herd Availability of IDT and/or IFN-γ results Availability of post-mortem data (when applicable)	Missing IDT or IFN-γ results Incomplete or inconsistent records Records not reliably linkable across databases

**Table 2:** Distribution of IDT results, IFN-γ results, and post-mortem findings in cattle subjected to bTB surveillance in the province of Palermo (Italy) during 2024–2025.

Variable	Category	2024 (%)	2025 (%)	Total (%)
IDT	Positive	57 (5.3)	36 (5.0)	93 (5.2%)
	Negative	1010 (93.8)	676 (93.4)	1686 (93.6%)
	Doubtful	10 (0.9)	12 (1.7)	22 (1.2%)
IFN-γ	Positive	79 (7.3)	84 (11.6)	163 (9.1%)
	Negative	922 (85.6)	500 (69.1)	1422 (79.0%)
	Inconclusive	76 (7.1)	140 (19.3)	216 (12.0%)
Post-mortem lesions *	Yes	26 (56.5)	38 (62.3)	64 (59.8%)
	No	20 (43.5)	23 (37.7)	43 (40.2%)

* Percentages for post-mortem lesions were calculated among slaughtered animals only (n = 46 in 2024; n = 61 in 2025; n = 107 overall).

**Table 3:** Year-specific agreement analysis between bTB diagnostic tests using Cohen’s kappa and prevalence-adjusted bias-adjusted kappa (PABAK), 2024–2025.

Year	Comparison	n	Po	κ (95% CI)	PABAK	Strength of Agreement *	N. Absolute Concordance
2024	IDT vs. IFN-γ	991	0.967	0.731 (0.640 to 0.821)	0.933	Substantial	958
2025	IDT vs. IFN-γ	572	0.909	0.453 (0.311 to 0.595)	0.818	Moderate	520
2024	IDT vs. Lesions	43	0.488	−0.073 (−0.386 to 0.241)	−0.023	Poor	21
2025	IDT vs. Lesions	61	0.459	−0.086 (−0.338 to 0.165)	−0.082	Poor	28
2024	IFN-γ vs. Lesions	46	0.587	0.056 (−0.269 to 0.381)	0.174	Slight	27
2025	IFN-γ vs. Lesions	51	0.549	−0.039 (−0.354 to 0.276)	0.098	Poor	28
2024	IDT vs. IFN-γ (slaughtered subgroup) †	43	0.674	−0.045 (−0.130 to 0.040)	0.349	Poor	29
2025	IDT vs. IFN-γ (slaughtered subgroup) †	52	0.385	−0.039 (−0.115 to 0.037)	−0.231	Poor	30

* Interpretation of Cohen’s Kappa (κ) according to [15]. The difference between n = 43 for IDT vs. Lesions and n = 46 for IFN-γ vs. Lesions in 2024 reflects the additional exclusion of three animals with doubtful IDT results from the former comparison. † Kappa estimates for the slaughtered subgroup are uninformative due to near-complete IFN-γ positivity (42/43 in 2024; 51/52 in 2025); results are reported for completeness only.

## Data Availability

The original contributions presented in this study are included in the article/Supplementary Material. Further inquiries can be directed to the corresponding authors.

## Supplementary Materials

The following supporting information can be downloaded at https://www.mdpi.com/article/10.3390/vetsci13090849/s1, Table S1: Contingency tables for pairwise agreement analyses between bTB diagnostic tests, by study year.

## Abbreviations

The following abbreviations are used in this manuscript:

bTB	Bovine tuberculosis
IDT	Intradermal tuberculin test
IFN-γ	Interferon-gamma release assay
