# Supplementary material for: Comparative Agreement of Intradermal Tuberculin Test and Interferon-Gamma Release Assay in Bovine Tuberculosis Surveillance in Sicily, Italy (2024–2025)
Source: Vet Sci. 2026 Aug 22;13(9):849. doi: 10.3390/vetsci13090849 (PMC13611586; doi:10.3390/vetsci13090849)
Supplement: Supplementary file 1 [file vetsci-13-00849-s001.zip › vetsci-4495127-supplementary.pdf]

Supplementary Table S1. Contingency tables for pairwise agreement analyses between bTB diagnostic tests, by study year.

2024 – IDT vs IFN-γ (n = 991)

IDT \ IFN-γ	Positive	Negative	Total
Positive	49	8	57
Negative	25	909	934
**Total**	**74**	**917**	**991**

2025 – IDT vs IFN-γ (n = 572)

IDT \ IFN-γ	Positive	Negative	Total
Positive	25	5	30
Negative	51	491	542
**Total**	**76**	**496**	**572**

2024 – IDT vs Lesions (n = 43)

IDT \ Lesions	Yes	No	Total
Positive	16	14	30
Negative	8	5	13
**Total**	**24**	**19**	**43**

2025 – IDT vs Lesions (n = 61)

IDT \ Lesions	Yes	No	Total
Positive	18	13	31
Negative	20	10	30
**Total**	**38**	**23**	**61**

2024 – IFN-γ vs Lesions (n = 46)

IFN-γ \ Lesions	Yes	No	Total
Positive	26	19	45
Negative	0	1	1

2024 – IFN-γ vs Lesions (n = 46)			
**IFN-γ \ Lesions**	**Yes**	**No**	**Total**
**Total**	**26**	**20**	**46**

2025 – IFN-γ vs Lesions (n = 51)			
**IFN-γ \ Lesions**	**Yes**	**No**	**Total**
Positive	28	22	50
Negative	1	0	1
**Total**	**29**	**22**	**51**
